# Cadmium Activates Multiple Signaling Pathways That Coordinately Stimulate Akt Activity to Enhance c-Myc mRNA Stability

**DOI:** 10.1371/journal.pone.0147011

**Published:** 2016-01-11

**Authors:** Jia-Shiuan Tsai, Cheng-Han Chao, Lih-Yuan Lin

**Affiliations:** 1 Institute of Molecular and Cellular Biology, and Department of Life Science, National Tsing Hua University, Hsinchu, Taiwan, ROC; 2 Division of Nephrology, Department of Internal Medicine, National Taiwan University Hospital Hsin-Chu Branch, Hsinchu, Taiwan, ROC; University of Louisville, UNITED STATES

## Abstract

Cadmium is a known environmental carcinogen. Exposure of Cd leads to the activation of several proto-oncogenes in cells. We investigated here the mechanism of c-Myc expression in hepatic cells under Cd treatment. The c-Myc protein and mRNA levels increased in dose- and time-dependent manners in HepG2 cells with Cd treatment. This increase was due to an increase in c-Myc mRNA stability. To explore the mechanism involved in enhancing the mRNA stability, several cellular signaling factors that evoked by Cd treatment were analyzed. PI3K, p38, ERK and JNK were activated by Cd. However, ERK did not participate in the Cd-induced c-Myc expression. Further analysis revealed that mTORC2 was a downstream factor of p38. PI3K, JNK and mTORC2 coordinately activated Akt. Akt was phosphorylated at Thr450 in the untreated cells. Cd treatment led to additional phosphorylation at Thr308 and Ser473. Blocking any of the three signaling factors resulted in the reduction of phosphorylation level at all three Akt sites. The activated Akt phosphorylated Foxo1 and allowed the modified protein to translocate into the cytoplasm. We conclude that Cd-induced accumulation of c-Myc requires the activation of several signaling pathways. The signals act coordinately for Akt activation and drive the Foxo1 from the nucleus to the cytoplasm. Reduction of Foxo1 in the nucleus reduces the transcription of its target genes that may affect c-Myc mRNA stability, resulting in a higher accumulation of the c-Myc proteins.

## Introduction

Cadmium (Cd) is an environmental pollutant and a known human carcinogen [1]. Upon uptake, Cd accumulates mainly in livers and kidneys and remains in the tissues for long periods of time [2]. Cd exposure causes various human cancers [3]. Cd induces and activates the metal-responsive transcription factor 1 (MTF-1) which regulates the expression of genes involved in metal homeostasis and oxidative stress [4]. Cd also stimulates the production of proto-oncogenes, such as c-Jun, c-Fos and c-Myc [5] and activates several signal transduction factors including p38, JNK, ERK, PI3K and Akt [6–9]. Thus, Cd exposure alters significantly the physiological functions of the cells.

c-Myc plays a crucial role in regulating cell proliferation, differentiation, cancer cell transformation and apoptosis [10]. Cellular quantity of c-Myc can be controlled at different levels. In addition to transcriptional control, the accumulation of c-Myc in cells can be altered by changing the mRNA or the protein stability [11]. The half-life of c-Myc is approximately 30 min and can be regulated by Raf-MEK-ERK and PI3K-Akt-GSK-3β, which are components of the Ras signaling pathway. The ERK and GSK-3β can phosphorylate c-Myc at Ser62 and Thr58, respectively. Reportedly, phosphorylation at Thr58 reduces while modification at Ser62 increases c-Myc stability [12]. Phosphorylation at Ser62 is a pre-requisite for the modification of Thr58. c-Myc with Thr58 phosphorylated can be recognized by ubiquitin E3 ligase SCF^FBW7^ and degraded by proteasome [13].

The half-life of the c-Myc mRNA is 15–30 min. The 3’-untranslated region (3’-UTR) of the c-Myc mRNA has AU-rich elements (AREs) that interacts with ARE-binding proteins (ARE-BP) [14]. Modulating the binding of *trans*-acting element with ARE alters the stability of the mRNA [15]. For example, binding with human antigen R (HuR) enhances c-Myc mRNA stability by masking the AU-rich sequence from the ribonuclease [16]. Conversely, binding of tristetraprolin (TTP) accelerates the removal of the poly-A tail and reduces the c-Myc mRNA stability [17]. In addition, the exon 3 of the c-Myc transcript contains unstable sequence, known as coding region instability determinant (CRD), which regulates the mRNA stability. The CRD-binding protein (CRD-BP) can protect the c-Myc mRNA from endoribonuclease cleavage [18].

Members of the forkhead box O (Foxo) family participate in the regulation of c-Myc gene expression [19]. Foxos are transcriptional factors that regulate the expression of miR-34c and miR-145 [20, 21]. Four isoforms, namely Foxo1 (FKHR), Foxo3 (FKHRL1), Foxo4 (AFX) and Foxo6, have been identified [22]. Foxo1, 3 and 4 are commonly found in mammals. Foxo1 has a high level expression in liver and adipose tissues. Foxo3 has a more significant level in hearts, brains, kidneys and spleens. Foxo4 and Foxo6 have highest expression in skeletal muscles and brains, respectively [23]. The activity of Foxo is modulated by the PI3K/Akt signaling factors. Akt phosphorylates three highly conserved serine/threonine residues on Foxo proteins and the modified product can thus interacts with the 14-3-3 proteins. 14-3-3 binds Foxo at the nuclear localization signal. This interaction reduces the DNA-binding activity of Foxo and stimulates the one-way translocation of Foxo from the nucleus to the cytoplasm [24].

Cd exposure enhances the production of reactive oxygen species in cells [25]. Upon Cd challenge, cells increase the synthesis of metallothionein (MT) and glutathione (GSH) via MTF-1-mediated gene transcription to sequester the toxic metal ions [26]. Simultaneously, cellular oxidative stress activates ERK which can phosphorylate c-Myc at Ser62. The modified c-Myc enhances the expression of γ-glutamylcysteine synthetase (γ-GCS) and results in elevated GSH synthesis [27]. Reportedly, c-Myc also binds to the MTIIA promoter to increase MT gene expression [28]. All of these responses may contribute to the cellular defense against Cd-induced toxicity. Despite this defense mechanism, excessive Cd exposure causes malignant effects. Cd is known to activate c-Myc proto-oncogene. However, the mechanism has not been explored. We report in this study the signaling pathway involved in the Cd-induced expression of c-Myc in hepatic cells.

## Materials and Methods

### Cell culture and chemicals

HepG2 cells were cultured at 37°C in Dulbecco’s Modified Eagle Medium supplemented with 10% heat-inactivated fetal bovine serum (FBS), 2 mM L-glutamine, 3.7 g/l sodium bicarbonate, 100 U/ml penicillin, and 100 μg/ml streptomycin in 5% CO_2_/95% air. Reagents for cell cultures were purchased from Invitrogen/GIBCO (Grand Island, NY, USA). Cadmium chloride, LY294002, PD98059 and Akt inhibitor IV were obtained from Merck (Darmstadt, Germany). SB202190 and SP600125 were purchased from AdooQ Bioscience (Irvine, CA, USA). Cycloheximide, actinomycin D and rapamycin were obtained from Sigma (St. Louis, MO, USA). Antibodies against phospho-Akt (Ser473), phospho-Akt (Thr308), phospho-Akt (Thr450), Akt, phospho-ERK1/2 (Thr202/Tyr204), ERK1/2, phospho-p38 (Thr180/Tyr182), phospho-JNK (Thr183/Tyr185), JNK, mTOR, phospho-p70 S6K (Thr389), phospho-Foxo1 (Thr24)/Foxo3a (Thr32), Foxo1 and phospho-NDRG1 (Thr346) were purchased from Cell Signaling Technology (Beverly, MA, USA). Antibodies against c-Myc, actin, p38, PCNA and anti-goat IgG were obtained from Santa Cruz Biotechnology (Santa Cruz, CA, USA). S6K and NDRG1 antibodies were obtained from GeneTex (Hsinchu, Taiwan). The antibody against Rictor was purchased from Novus Biologicals (Littleton, CO, USA). Alpha-tubulin and lamin B1 antibodies were obtained from Thermo Scientific (Waltham, MA, USA) and Abcam (Cambridge, MA, USA), respectively. OPTI-MEM I, various siRNAs and Lipofectamin^™^ RNAiMAX were purchased from Invitrogen (Carlsbad, CA, USA). X-tremeGENE HP was purchased from Roche (Mannheim, Germany). Horseradish peroxidase-conjugated anti-mouse, anti-rabbit IgG were from GE Healthcare. Enzymes and reagents for reverse transcription were obtained from Fermentas (Vilnius, Lithuania). Restriction enzymes were purchased from New England Biolabs (Ipswich, MA, USA). Protein molecular weight marker was from SMOBIO (Hsinchu, Taiwan).

### Plasmid construction

Genomic DNA extracted from HepG2 cells was used as template for the amplification of the c-Myc (-1202 to +19) promoter. The primer sequences are listed in S1 Table. The product from polymerase chain reaction (PCR) was isolated electrophoretically, eluted from agarose gel then cloned into the PGL3-basic luciferase plasmid (Promega, Madison, WI, USA). The GFP-Foxo1 wild type (WT) and GFP-Foxo1 3A (Foxo1 with alanines substitutions at Thr24, Ser256 and S319, and acts as constitutively active Foxo1) expression plasmids were kindly provided by Dr. Shen-Liang Chen (Department of Life Sciences, National Central University, Taiwan).

### Transfection and reporter gene assay

HepG2 cells (1.7 x 10^4^) were seeded in 96-well culture dishes overnight. Before transfection, 0.25 μg of DNA (0.2 μg of c-Myc reporter plasmid and 0.05 μg of pcDNA3-eGFP) was mixed with 20 μl of OPTI-MEM I. Then, 1 μl of X-tremeGENE HP was added to the DNA/ OPTI-MEM I mixture prepared above. The DNA mixture was set for a further 30 min before transfecting the cells. The transfected cells were cultured for 48 h followed by various treatments. Luciferase assays were carried out using the Promega luciferase assay system according to the manufacturer’s instructions on a multilabel plate reader (Wallac 1420 VICTOR^2^, Perkin Elmer, Foster City, CA, USA).

For siRNA transfection, 5 x 10^5^ HepG2 cells were seeded in 6-well plates with 2.5 ml of antibiotics-free medium overnight until the cells were 30% to 50% confluent. Transfections were conducted using 150 pmol of siRNA and Lipofectamine™ RNAiMAX following the manufacturer’s instructions. Cells were cultured for another 48 h before various treatments.

### Real-time PCR

Total RNA was extracted with TRIzol^®^ Reagent (Invitrogen) following the procedures provided by the manufacturer. The extracted RNA (1 μg) was reverse-transcribed with a RevertAid™ First Strand cDNA Synthesis Kit (Fermentas). The resulting complementary DNA was used for quantitative real-time PCR (qPCR) with SYBR^®^ Green PCR Master Mix (Applied Biosystems, Foster City, CA, USA) on an Applied Biosystems StepOnePlus™ Real-Time PCR System. Primers used in the reactions are listed in S1 Table. The expression of human glyceraldehyde 3-phosphate dehydrogenase (GAPDH) was determined in each sample and used as an internal control. Expression of c-Myc mRNA was compared on the basis of equivalent GAPDH transcripts.

### Immunoblotting analysis

Cells were harvested by centrifugation then resuspended in three volumes of extraction buffer (20 mM HEPES, pH 7.9, 0.4 M NaCl, 1 mM PMSF, 50 mM NaF, 0.5 mM Na_3_VO_4_, 2 μg/ml aprotinin, 5 μg/ml leupeptin, 1 μg/ml pepstatin, and 0.5% Nonidet P-40). The tubes were incubated on ice for 10 min before rocking vigorously for 15 sec at 4°C. The procedure was repeated four times. The cell lysates were separated by centrifugation at 16,000 x g for 10 min, and the supernatants were loaded on an 8% SDS-polyacrylamide gel (5% stacking gel) for electrophoretic separation. The separated proteins were transferred onto a PVDF membrane (GE Healthcare, Milwaukee, WI, USA) using a transfer cell (Bio-Rad, Hercules, CA, USA). The membrane was pre-hybridized in TBST buffer (150 mM NaCl, 10 mM Tris, pH8.0, 0.1% Tween-20) with 5% skim milk for 1 h, and then transferred to a solution containing 5% skim milk and primary antibodies. After shaking at 4°C overnight, the membrane was washed three times with TBST buffer then submerged in 5% skim milk containing horseradish peroxidase-conjugated secondary antibodies for 1 h. After washing three times with TBST buffer and rinsing once with TBS buffer (TBST buffer without Tween-20), the membrane was developed by the ECL system (PerkinElmer) and exposed to X-ray film for visualization. The intensity of the protein bands on the X-ray films were quantified using the UN-SCAN-IT gel analysis software (version 6) and compared to that of the loading control in each sample in the calculations.

### Separation of cytosolic and nuclear fractions

Harvested cells were resuspended in a hypotonic buffer (10 mM HEPES pH 7.9, 10 mM KCl, 0.5 mM PMSF, 50 mM NaF, 0.5 mM Na3VO4, and mixed protease inhibitors) and incubated on ice for 15 min before Nonidet P-40 was added to a final concentration of 0.5%. After shaking vigorously for 10 sec, the homogenate was centrifuged at 6,000 rpm for 10 min and the supernatant was removed and designated as the cytosolic fraction. The pellet was resuspended in extraction buffer (20 mM HEPES pH 7.9, 0.4 M NaCl, 1 mM PMSF, 50 mM NaF, 0.5 mM Na3VO4, and mixed protease inhibitors) and rocked vigorously at 4°C for 40 min. The mixture was centrifuged for 10 min and the supernatant was collected and designated as the nuclear fraction.

### Statistical analysis

All statistical analyses were performed using two-tailed Student’s t-tests (Microsoft Excel software, Microsoft Corporation, Washington, USA). All *p* values <0.05 were considered statistically significant.

## Results

**Fig 1:**
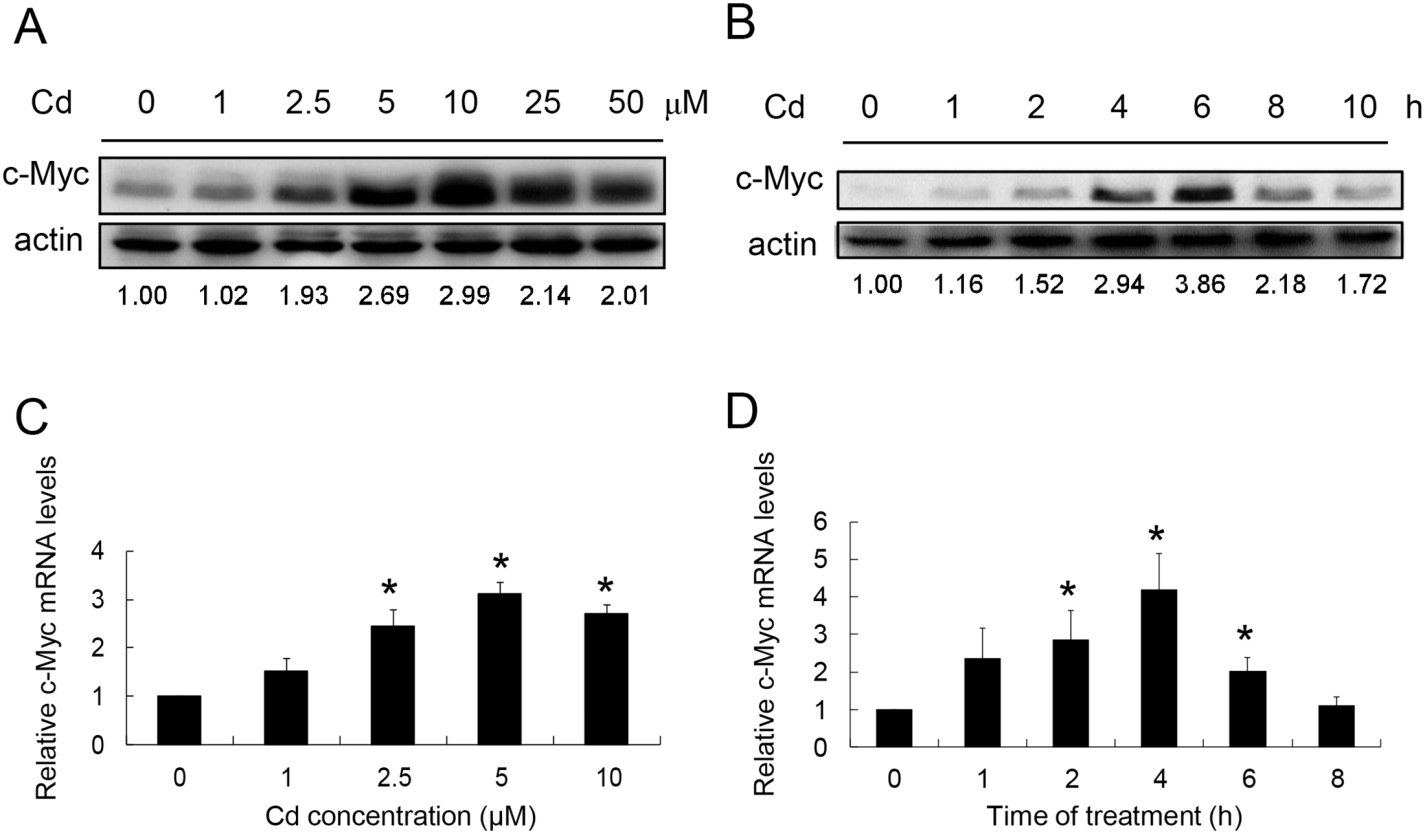
Effect of Cd on c-Myc expression in HepG2 cells. (A) Cells were treated with various concentrations of Cd for 4 h and cellular c-Myc was quantified by Western blotting. (B) Cells were treated with 5 μM Cd for various time intervals and cellular c-Myc was quantified by Western blotting. A minor band with higher molecular weight can be observed and it is the isofom of c-Myc. Numbers underneath the Wester blotting represent the amount of c-Myc relative to that of the loading control (actin). The dose-response (C) and time-course (D) studies were repeated and the c-Myc mRNA was quantified by real-time PCR and normalized to that of the GAPDH mRNA. Each value represents a mean ± standard deviation of three samples. Asterisks (*) indicate significant difference (*p* < 0.05) as compared to that of the control (untreated) sample.

A dose-dependent study was conducted to examine whether Cd induces c-Myc expression in HepG2 cells. Cells were treated with 1–50 μM Cd for 4 h and the amounts of c-Myc proteins were quantified. Fig 1A shows that c-Myc increases with Cd administration. The protein level reaches the highest level at 10 μM treatment then declines at higher Cd concentrations. Time-course study was then performed. Cells were treated with 5 μM Cd for various time intervals. The results show that c-Myc increased significantly after 4 h treatment and reached a maximum level at 6 h (Fig 1B). The c-Myc gene expression is also examined with Cd treatment. As shown in Fig 1C, c-Myc mRNA increased in a dose-dependent manner with the highest level at 5 μM Cd treatment. Under this condition, the c-Myc mRNA has the highest level of accumulation after 4 h of Cd treatment (Fig 1D). These results indicate that Cd treatment enhances the level of c-Myc mRNA resulting in a higher level of protein accumulation in the cells.

**Fig 2:**
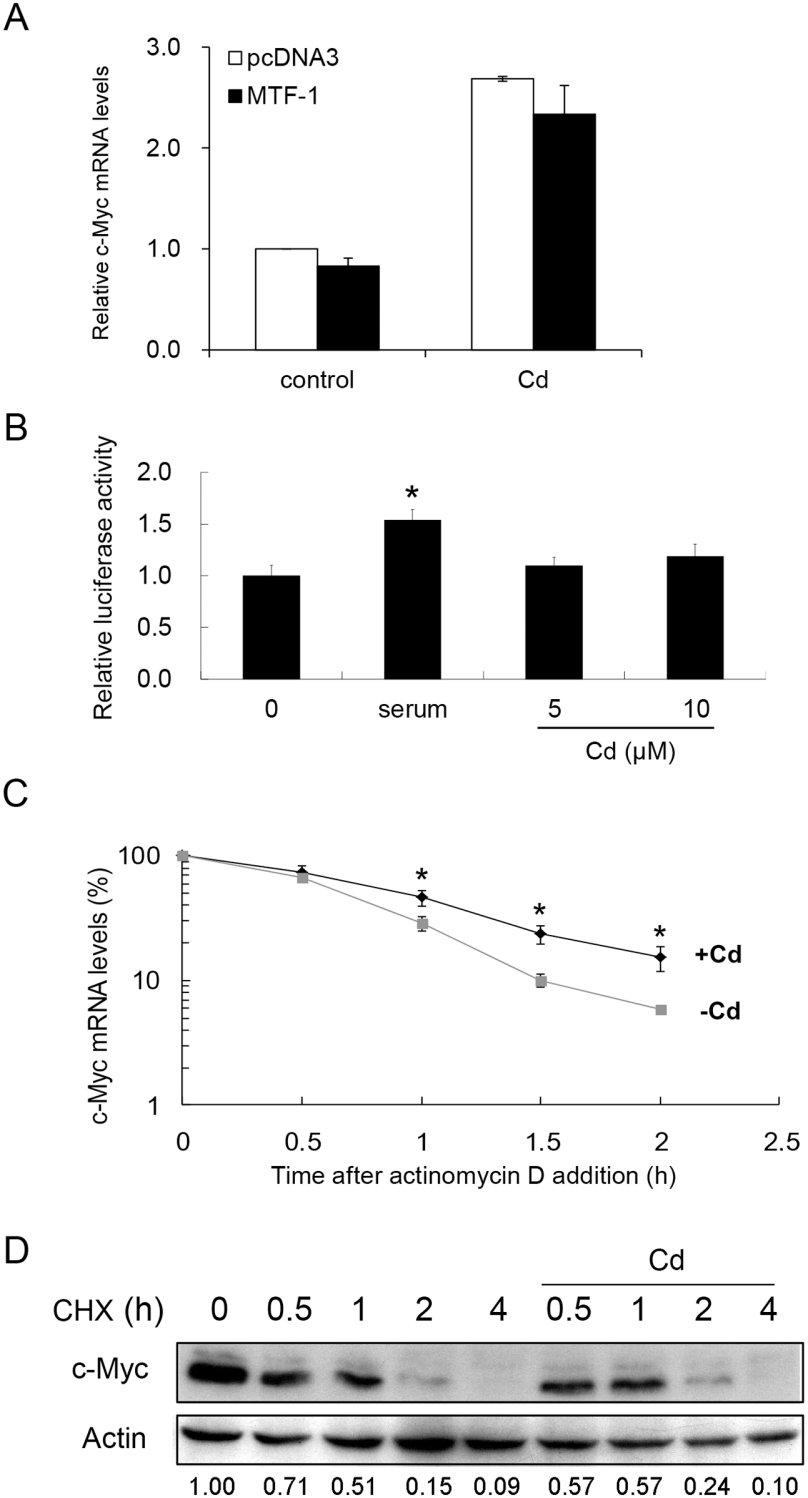
Analysis of factors that modulate the cellular c-Myc level after Cd administration. (A) MTF-1 was over-expressed in HepG2 cells. After adding 0 or 5 μM Cd for 4 h, cells were harvested and cellular c-Myc mRNA was quantified by real-time PCR. (B) A reporter plasmid carrying the promoter region of the c-Myc gene (-1202 to +19) was transfected to HepG2 cells and cultured in serum-free medium for 24 h. The cells were then treated with 10% serum for 12 h or Cd at the concentrations specified for 4 h. Luciferase activities from the samples were analyzed. Asterisk (*) indicates significant difference (*p* < 0.05) as compared to that of the untreated control. (C) HepG2 cells were pretreated with 0 or 5 μM Cd for 2 h before adding actinomycin D to a final concentration of 5 μg/ml. Cells were harvested at various time intervals and c-Myc mRNA was quantified by real-time PCR. Asterisks (*) indicate significant differences (*p* < 0.05) between the paired samples (the same time interval). Each value represents a mean ± standard deviation of three samples. (D) HepG2 cells were pretreated with 20 μM cyclohexamide (CHX) for 15 min before adding 0 or 5 μM Cd. Cells were cultured for additional 4 h. Samples were removed at various time intervals and cellular c-Myc was examined by Western blotting. Actin was used as a loading control to normalize the amount of sample applied for analysis. Numbers underneath the Western blotting represent the amount of c-Myc relative to that of the loading control (actin).

We investigated the involvement of MTF-1 in this process. As shown in Fig 2A, over-expression of MTF-1 in the cells did not alter the c-Myc mRNA level either in the absence or presence of Cd.

A reporter plasmid with the upstream flanking region of the c-Myc gene (as promoter) was constructed and transfected into HepG2 cells. Cd did not activate the reporter activity in the transfected cells. Serum treatment that has been known to activate the c-Myc promoter was included as positive control in the experiments (Fig 2B). These results suggest that Cd does not act directly on the c-Myc gene expression at the transcriptional level.

Reportedly, the half-life of the c-Myc mRNA increases with arsenic treatment [29]. We therefore examined the mRNA stability after Cd administration. Cells were treated with or without Cd in the presence of actinomycin D, and harvested at various time intervals for c-Myc transcripts quantification. Fig 2C shows that the level of c-Myc mRNA differs significantly 1 h after Cd treatment. The Cd-treated cells had a higher level of c-Myc mRNA than that of the untreated cells.

The c-Myc protein stability was also analyzed. Cells pretreated with cyclohexamide were incubated with 5 μM Cd for various time intervals. Cells were then harvested and the c-Myc levels in the cells were quantified by Western blotting. The results show that Cd is ineffective in changing the c-Myc level in cells pretreated with cyclehexamide (Fig 2D). These results show clearly that Cd treatment stabilizes the c-Myc mRNA and consequently elevates the protein level without changing the protein turnover rate.

**Fig 3:**
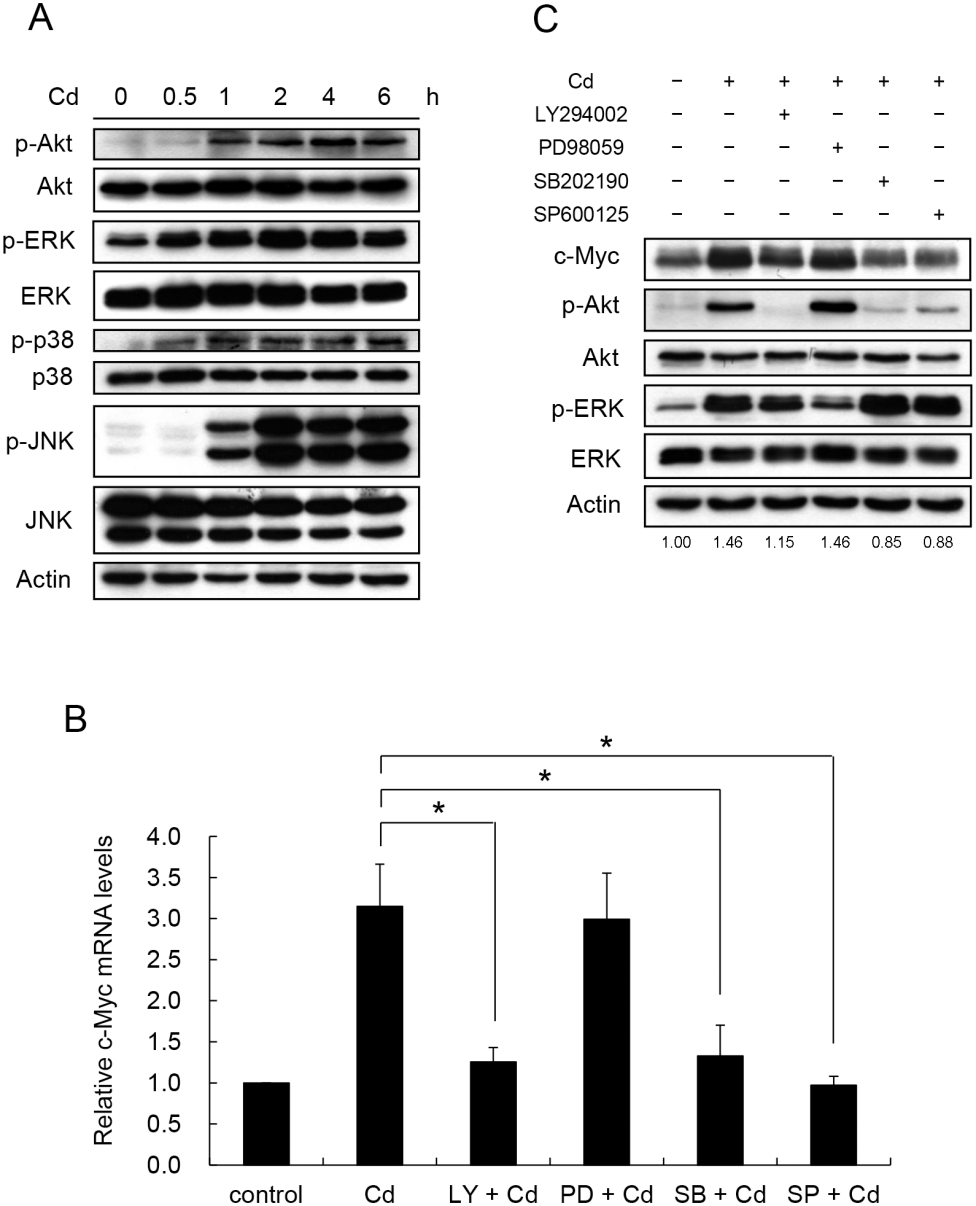
Cd activates PI3K, p38 and JNK signaling factors to increase c-Myc mRNA and protein. HepG2 cells were treated with 5 μM Cd for 6 hours. Samples were removed at various time intervals and the phosphorylation of the signaling factors was determined by Western blotting. (B) Cells were treated with 50 μM of inhibitor for PI3K (LY294002), ERK (PD98059), p38 (SB202190) and JNK (SP600125) 1 h prior to the addition of 5 μM Cd. Cells were cultured for additional 4 h. Total RNA was extracted and the c-Myc mRNA was quantified by real-time PCR. Each value represents a mean ± standard deviation of three samples. Asterisks (*) indicate significant differences (*p* < 0.05) between the paired samples. (C) Cellular c-Myc after various inhibitor treatments as in (B) was analyzed by Western blotting. Phospho-Akt was detected by antibodies specific for the modification at Ser473. Phosphate proteins are designated with ‘p-‘ in front of the protein name. LY: LY294002; PD: PD98059; SB: SB202190; SP: SP600125. Numbers underneath the Western blotting represent the amount of c-Myc relative to that of the loading control (actin).

We speculated that the change in c-Myc mRNA stability is achieved by factors in signaling pathways activated by Cd treatment. To explore the mechanism, the activities of a few selected signaling factors were examined. Reportedly, Cd activates Akt, ERK, p38, or JNK in different cell types [6–9]. In HepG2 cells, Akt, ERK, p38, and JNK were phosphorylated (activated) within 2 h of Cd treatment (Fig 3A). To investigate which or all of these factors contribute to the increase in c-Myc mRNA, inhibitor for each factor was applied to the cells. Since Akt is activated via the PI3K/PDK1 pathway [30], the participation of PI3K in this signaling pathway is expected. Cells were treated with SB202190 (p38 inhibitor), LY294002 (PI3K inhibitor), PD98059 (ERK inhibitor) or SP600125 (JNK inhibitor) 1 h before Cd administration. Fig 3B shows that Cd-induced c-Myc mRNA increase was attenuated by the PI3K, p38 and JNK inhibitors but not the ERK inhibitor. The differences were also reflected at the protein level. Cd treatment increased the c-Myc level and the increase was blocked in the presence of SB202190, LY294002 and SP600125, but not PD98059 (Fig 3C). This finding indicates that ERK is not involved in the Cd-induced c-Myc expression. Noticeably, SB202190, LY294002 and SP600125 also reduced Akt activity. Because PI3K and Akt activities increased with Cd treatment, the upstream regulator of PI3K (PTEN) [31] was also examined. Cd treatment did not affect the level of PTEN in the cells, indicating that Cd possibly activates PI3K directly (data not shown).

To further investigate whether the signaling factors are activated by Cd in a parallel or sequential manner, the effects of these inhibitors on the activity of the signaling factors were examined. Noticeably, the PI3K inhibitor reduced Akt activity and the quantity of c-Myc. However, ERK inhibitor had no effect on Akt activity and c-Myc level (Fig 3C). The p38 and JNK inhibitors blocked the kinase activity without changing the phosphorylation level of the target proteins. Therefore, SB202190 and SP600125 did not change the phosphorylation level of p38 and JNK, respectively (data not shown). But these inhibitors diminished the phosphorylation of Akt and the level of c-Myc (Fig 3C). These results suggest that p38, JNK and PI3K were concurrently activated by Cd and Akt is the key and common target for these factors in the induction of c-Myc.

**Fig 4:**
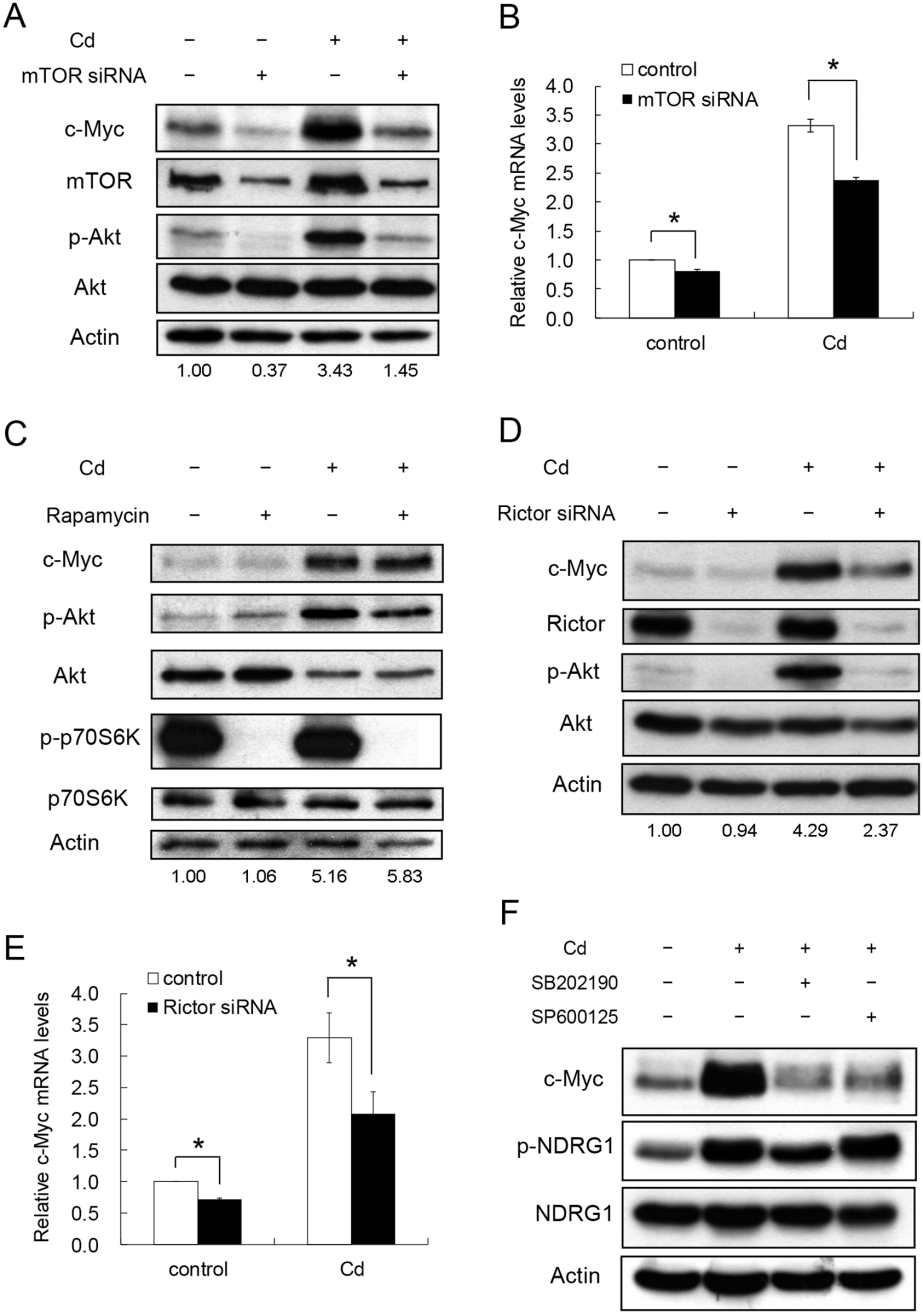
Participation of mTORC2 in the p38 signaling pathway to regulate c-Myc expression after Cd exposure. HepG2 cells were treated with mTOR siRNA to reduce total mTOR expression then cultured in the presence or absence of 5 μM Cd for 4 h. Cells were harvested for Western blotting (A) and real-time PCR analysis (B). (C) Cells were treated with 100 nM rapamycin 1 h prior to the addition of 5 μM Cd then cultured for additional 4 h. Cellular proteins were extracted for Western blotting. (D) Cells were transfected with 50 nM control or Rictor siRNA. After treating with 5 μM Cd for 4 h, cells were harvested. Cell extracts were prepared for Western blotting. (E) c-Myc mRNAs from samples prepared as in (D) were quantified by real-time PCR. (F) Cells were treated with 50 μM p38 (SB202190) or JNK (SP600125) inhibitor for 1 h followed by the addition of 5 μM Cd. Cells were cultured for an additional 4 h. Cell extracts were prepared for Western blotting. Phospho-Akt was detected by the antibodies specific for the modification at Ser473. Actin was used as a loading control for immunoblotting. Phosphate proteins are designated with ‘p-‘ in front of the protein name. For RNA analysis, each value represents a mean ± standard deviation of three samples. Asterisks (*) indicate significant differences (*p* < 0.05) between the paired samples. Numbers underneath the Western blotting represent the amount of c-Myc relative to that of the loading control (actin).

mTOR is one of the upstream regulators of Akt activity [32]. We examined the role of mTOR in controlling the c-Myc level and the Akt activity under Cd-induced condition. Cells were transfected with mTOR siRNA to knock-down the gene expression. As shown in Fig 4A, siRNA treatment reduced the mTOR, phospho-Akt and c-Myc levels with or without Cd administration. To investigate whether the effect is controlled at the mRNA level, the c-Myc transcripts were quantified. Fig 4B shows that c-Myc mRNA reduced with the mTOR siRNA treatment. This result demonstrates clearly that c-Myc expression is tightly monitored by the mTOR-Akt pathway.

The integral activity of mTOR derives from the complex form of the protein associated with other components. Two complex forms, namely mTORC1 and mTORC2, are found to have different activities [33]. The phosphorylation of the p70S6K protein was a good indicator of the mTORC1 activity [34], and the reaction can be specifically inhibited by rapamycin [35]. Rapamycin effectively blocked the p70S6K phosphorylation in the presence and absence of Cd (Fig 4C). However, the c-Myc level was not altered by the rapamycin treatment. This result implies that mTORC1 does not regulate the c-Myc expression. Rictor is the key protein in the mTORC2 complex and regulates its activity [33]. The expression of Rictor was knocked-down with siRNA. Fig 4D shows that the level of Rictor was not affected by Cd treatment but suppressed by the siRNA. Remarkably, the siRNA treatment abolished the Cd-activated Akt activity and decreased the c-Myc level. In addition, Rictor siRNA can significantly reduce the levels of c-Myc transcripts with or without Cd treatment (Fig 4E). The same effect was noted in Cd-treated cells with the addition of actinomycin D (S1 Fig). These results indicate that mTORC2 modulates Akt activity and controls the c-Myc level in the cells.

To further address whether the mTORC2 activity was regulated by upstream signaling factors, the activity of SGK1 (serum and glucocorticoid induced protein kinase 1) was used as an indicator of the assay. Similar to Akt, SGK1 is a downstream target of mTORC2. Activated SGK1 phosphorylates NDRG1 at Thr342 and this modification can be utilized as a reliable marker for mTORC2 signaling [36]. Fig 4F shows that, the p38 inhibitor (SB202190) decreased the level of phosphorylated NDRG1 in Cd-treated cells. This phenomenon was not observed with the addition of JNK inhibitor (SP600125). The result demonstrates that p38 is the upstream regulator of mTORC2 in modulating the c-Myc expression after Cd administration.

**Fig 5:**
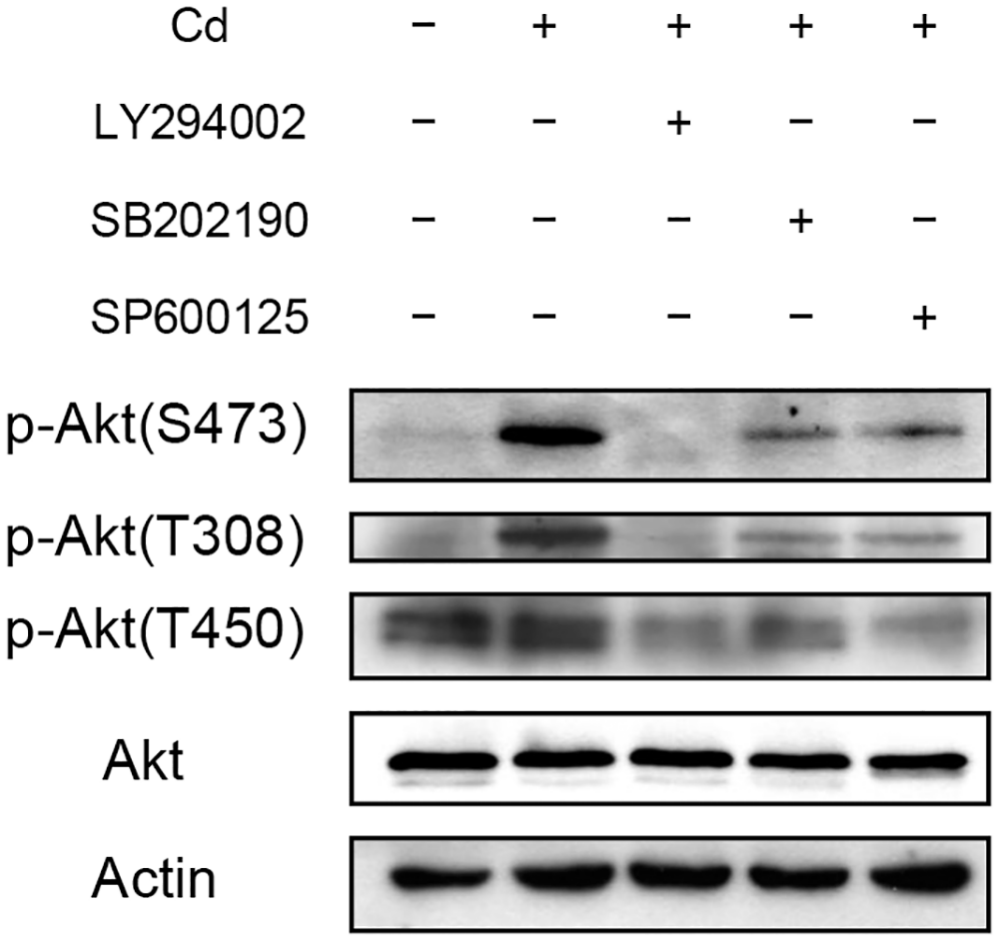
Effect of Cd on Akt phosphorylaion at different sites. HepG2 cells were treated with 50 μM of PI3K ((LY294002), p38 (SB202190) or JNK (SP600125) inhibitor 1 h prior to the addition of 5 μM Cd. Cells were cultured for additional 4 h then harvested and prepared for Western blotting. Antibodies raised specifically against phosphorylation at Thr308 [p-Akt(T308)], Thr450 [p-Akt(T450)] or Ser473 [p-Akt(S473)] of Akt were used for the analysis. Actin was used as a loading control for immunoblotting.

Thr308, Thr450 and Ser473 on Akt can be phosphorylated by PDK1 (target of PI3K), JNK and mTORC2, respectively. Reportedly, Thr450 is modified by JNK then further modifications at Thr308 and Ser473 by PDK1 and mTORC2 allow Akt to gain full activity [37]. We examined whether a sequential change of the modifications on Akt occurred after Cd treatment. As shown in Fig 5, significant amount of phosphorylated Thr450 existed in untreated cells. Administration of Cd did not alter the modification level of Thr450, but greatly increased the phosphorylation at Thr308 and Ser473. Addition of any one of the PI3K, p38 or JNK inhibitors to the Cd-treated cells resulted in a reduction of phosphorylation level at all three sites. This result indicates that modification of these sites after Cd treatment is important to gain the integral function of Akt that leads to an increase in c-Myc.

**Fig 6:**
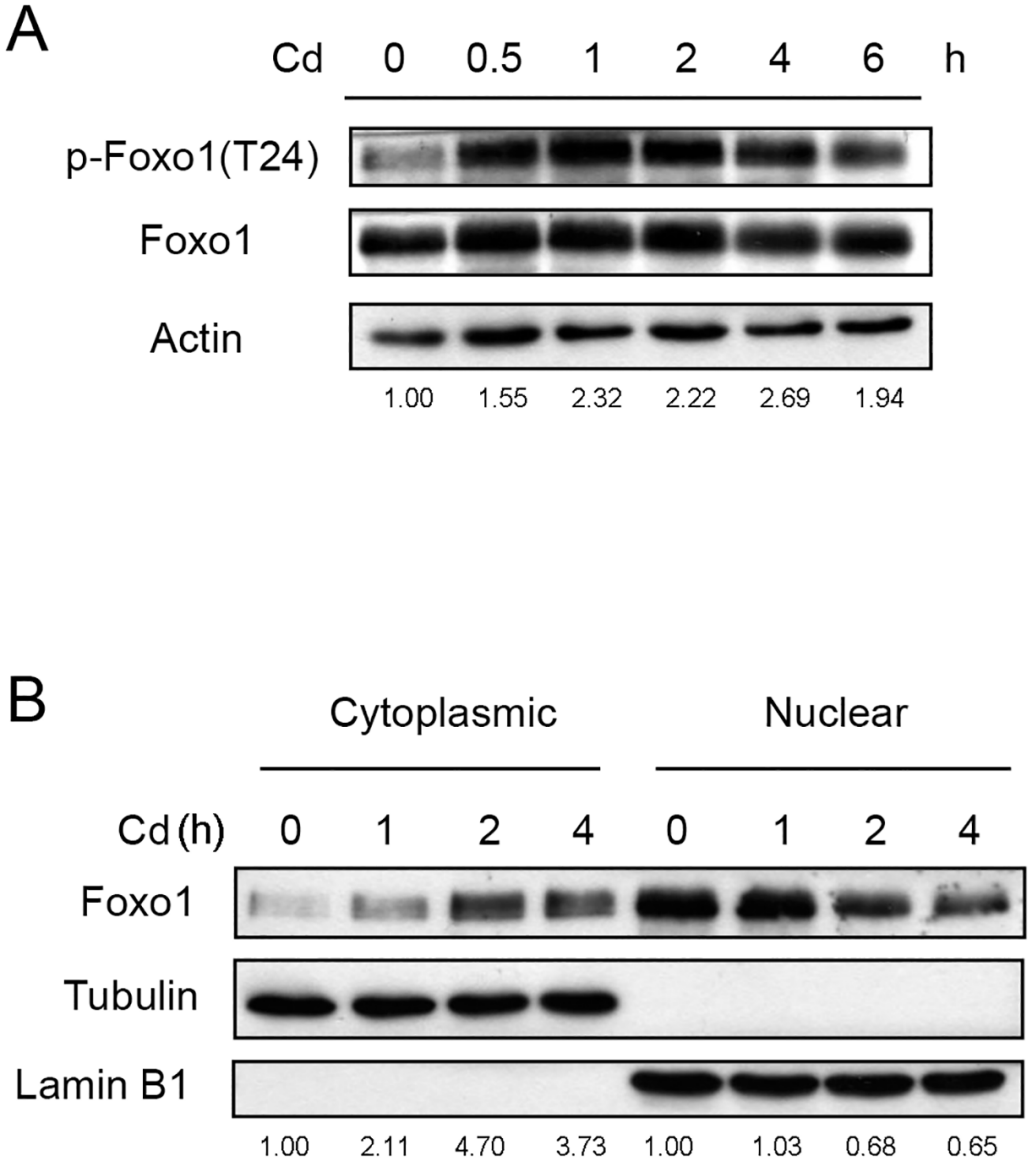
Cd stimulates the phosphorylation of Foxo1 and translocations the protein from the nucleus to the cytoplasm. (A) HepG2 cells were treated with 5 μM Cd for various time intervals and the levels of Foxo1 and phospho-Foxo1 [p-Foxo1(T24)] were analyzed by Western blotting. Numbers underneath the Western blotting represent the amount of phospho-Foxo1 relative to that of the loading control (actin). (B) Cells were treated with 5 μM Cd for various time intervals. Cells were harvested and cytosolic and nuclear fractions were prepared for Western blotting. Numbers underneath the Western blotting represent the amount of Foxo1 relative to that of the loading control (tubulin for cytoplasmic proteins and lamin B1 for nuclear proteins).

It has been established that the transcription factor Foxo can be phosphorylated by Akt. The modified protein then translocates from the nucleus to the cytoplasm [24]. This movement results in a reduction of nuclear Foxo level and may alter downstream gene expression. The modification of Foxo after Cd treatment was thus examined. Antibodies used herein can detect both Foxo1 and Foxo3, but Foxo1 can be differentiated from Foxo3 by its molecular weight. However, only Foxo1 was detected in HepG2 cells. As shown in Fig 6A, the amount of phosphorylated Foxo1 increased within 30 min of Cd administration and declined gradually upon prolonged treatment. The cellular distribution of Foxo1 was subsequently analyzed. Fig 6B shows that the amount of Foxo1 increased in the cytosolic fraction with time. On the contrary, the Foxo1 content decreased in the nuclear fraction after Cd treatment. Because phosphorylation at Thr24 is required for nuclear Foxo1 to translocate, the results indicate that Cd administration causes a trafficking of Foxo1 from the nucleus to the cytoplasm via the modification at Thr24.

**Fig 7:**
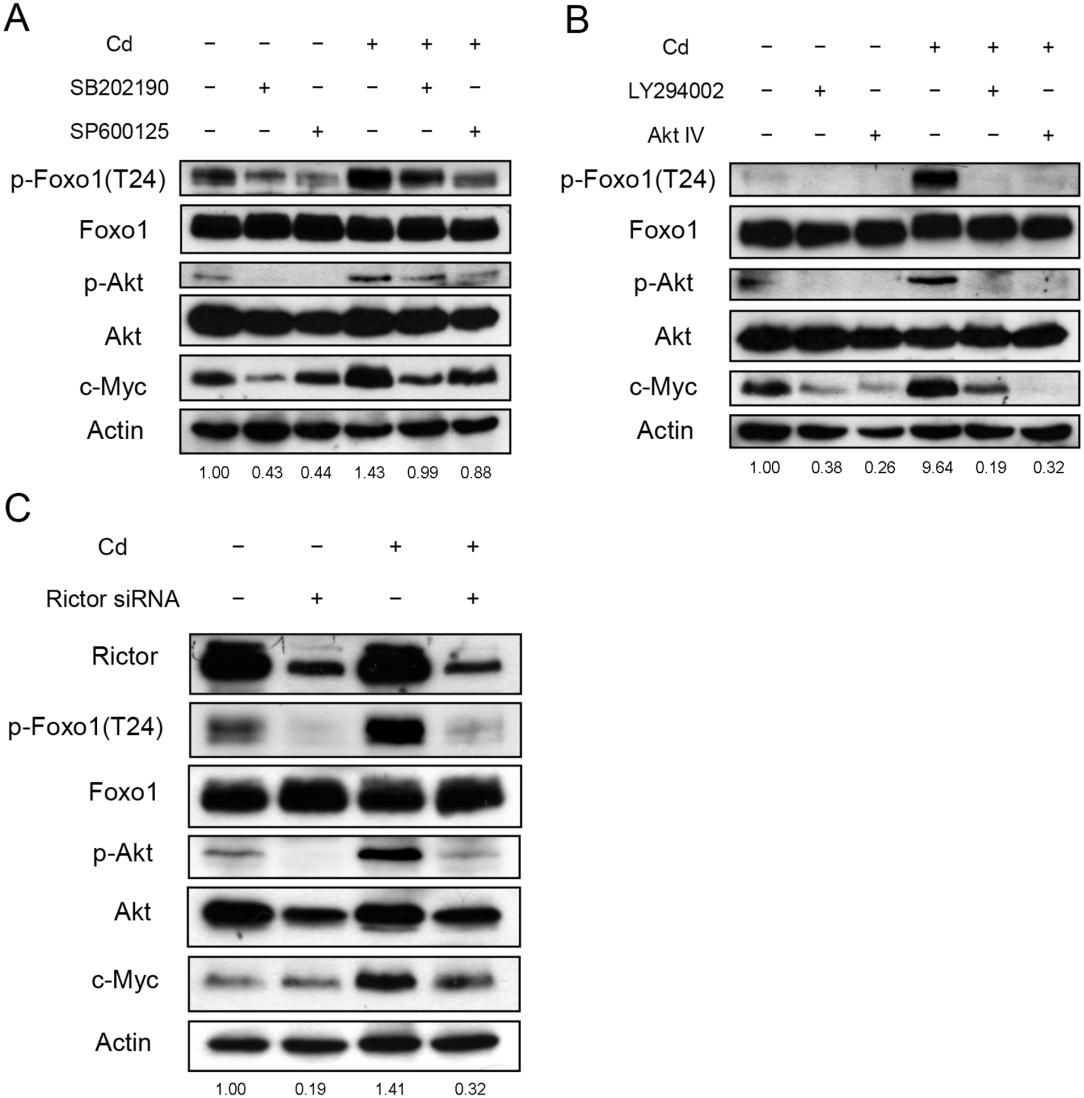
Phosphorylation of Foxo1 is regulated by PI3K, p38 and JNK. (A) HepG2 cells were pre-treated with 50 μM SB202190 (p38 inhibitor) or SP600125 (JNK inhibitor) for 1 h; (B) Cells were pre-treated with 50 μM LY294002 (PI3K inhibitor) or 2 μM Akt IV (Akt inhibitor) for 1 h; (C) Cells were transfected with 50 nM control or Rictor siRNA. Cells from (A)—(C) were further treated with 5 μM Cd for 1 h then harvested. Cell extracts were prepared for immunoblotting and quantification of c-Myc, Foxo1, phospho-Foxo1 [p-Foxo1(T24)], Akt and Akt phosphorylated at Ser473 (p-Akt). Numbers underneath the Western blotting represent the amount of phospho-Foxo1 relative to that of the loading control (actin).

In order to establish the signaling pathway for the Foxo1 phosphorylation, cells were treated with various inhibitors 1 h before Cd addition. Cells were harvested 1 h after Cd treatment and cellular proteins were analyzed. JNK and p38 inhibitors reduced the levels of phospho-Foxo1, phospho-Akt and c-Myc (Fig 7A). The PI3K inhibitor also blocked the phosphorylation of Foxo1 (Fig 7B). Similarly, phospho-Akt and c-Myc levels decreased under the treatment. However, the phospho-Foxo1 level was not altered when ERK inhibitor (PD98059) was administered (data not shown). In addition, the role of mTORC2 was investigated. As shown in Fig 7C, knock-down of Rictor abolished the phosphorylation of Foxo1, and reduced the levels of phospho-Akt and c-Myc. These results indicate that Akt is the common factor that receives various upstream signals to modify the Foxo1 after Cd addition.

**Fig 8:**
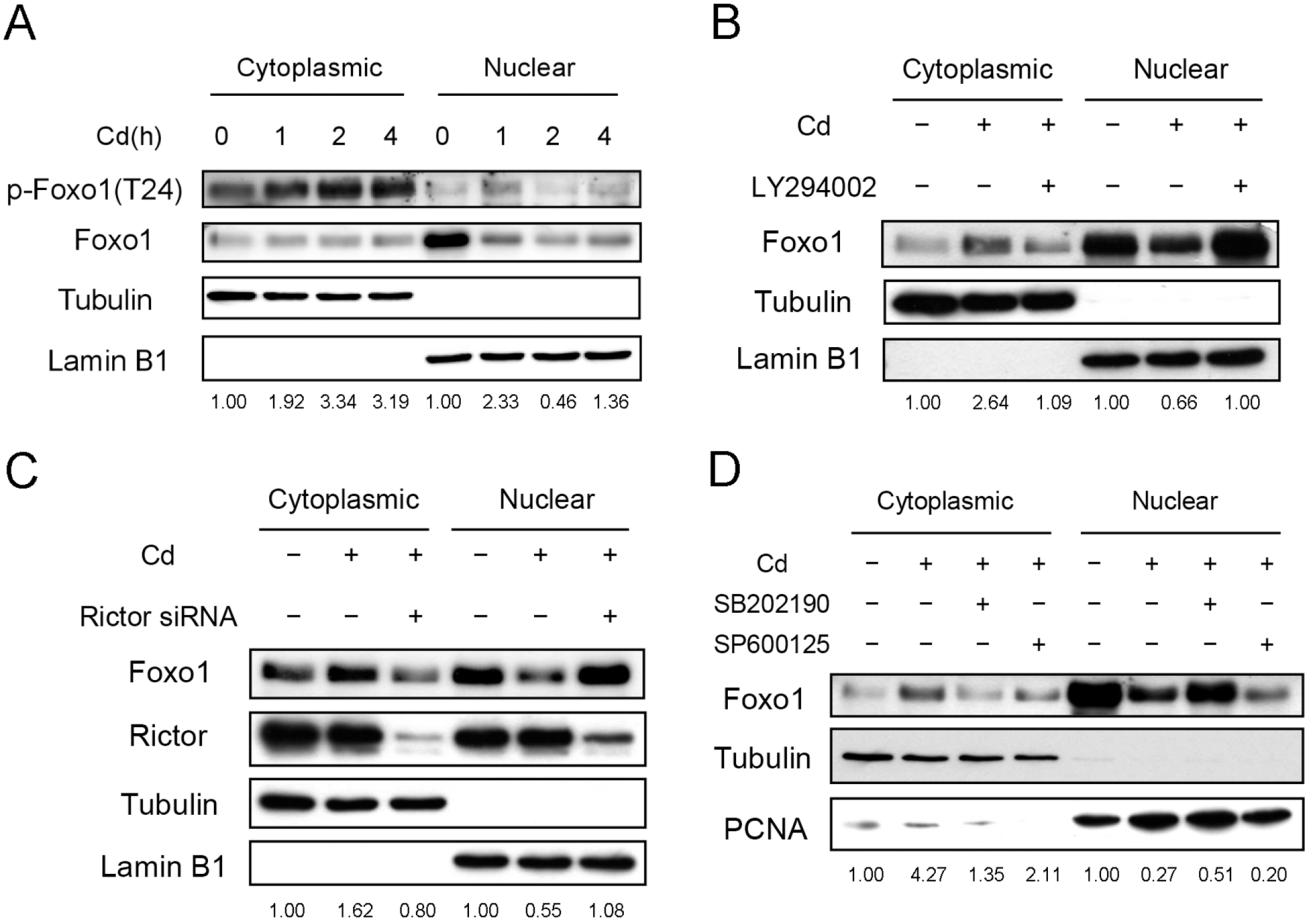
Translocation of Foxo1 is regulated by PI3K, p38 and JNK. (A) HepG2 cells were treated with 5 μM Cd for various time intervals. Cells were harvested and cytosolic and nuclear fractions were prepared for Western blotting. (B) Cells were pre-treated with 50 μM LY294002 for 1h; (C) Cells were transfected with 50 nM control or Rictor siRNA; (D) Cells were pre-treated with 50 μM SB202190 or SP600125 for 1 h. Cells from (B)–(D) were further treated with 5 μM Cd for 4 h. Cell extracts were separated into cytosolic and nuclear fractions for Western blotting. Numbers underneath the Western blotting represent the amount of phospho-Foxo1 (A) or Foxo1 (B–D) relative to that of the loading control (tubulin for cytoplasmic proteins and lamin B1 or PCNA for nuclear proteins).

Since phosphorylation of Foxo1 translocates the protein from the nucleus to the cytoplasm [24], the cellular localization of phosphorylated Foxo1 was examined after treating Cd for various time intervals. As shown in Fig 8A, phospho-Foxo1 level in the cytoplasm increased with time. On the contrary, nuclear phospho-Foxo1 increased within 1 hour of Cd treatment and declined afterward. Possibly, nuclear Foxo1 was phosphorylated shortly after Cd treatment. The phospho-Foxo1 then moved out of the nucleus. The phosphorylation of Foxo1 was apparently modulated by nuclear Akt since the level of phospho-Akt increased in nucleus after Cd treatment (S2 Fig). The cellular distribution of Foxo1 was also investigated in cells treated with PI3K inhibitor. Fig 8B shows that Cd treatment increased cytoplasmic Foxo1 level and the increase was attenuated by the PI3K inhibitor. Concurrently, nuclear Foxo1 decreases with Cd administration and the decrease was averted by the PI3K inhibitor. The results are in agreement with the observation that the PI3K inhibitor prohibited the phosphorylation of Foxo1 (Fig 7B), hence blocking the translocation from the nuclear to the cytoplasm. Foxo1 distribution was also examined with the knock-down of Rictor expression. Cells were transfected with the Rictor siRNA and cellular distribution of Foxo1 was analyzed after Cd treatment. Fig 8C shows that Rictor knock-down blocks the translocation of Foxo1. This finding demonstrates again that mTORC2 is the upstream signaling factor of Foxo1. Furthermore, the Foxo1 distribution is studied in cells administered with p38 or JNK inhibitor. As shown in Fig 8D, addition of p38 inhibitor held the Foxo1 in the nucleus. However, JNK inhibitor caused a dramatic reduction of nuclear Foxo1 without increasing the amount of cytoplasmic Foxo1.

**Fig 9:**
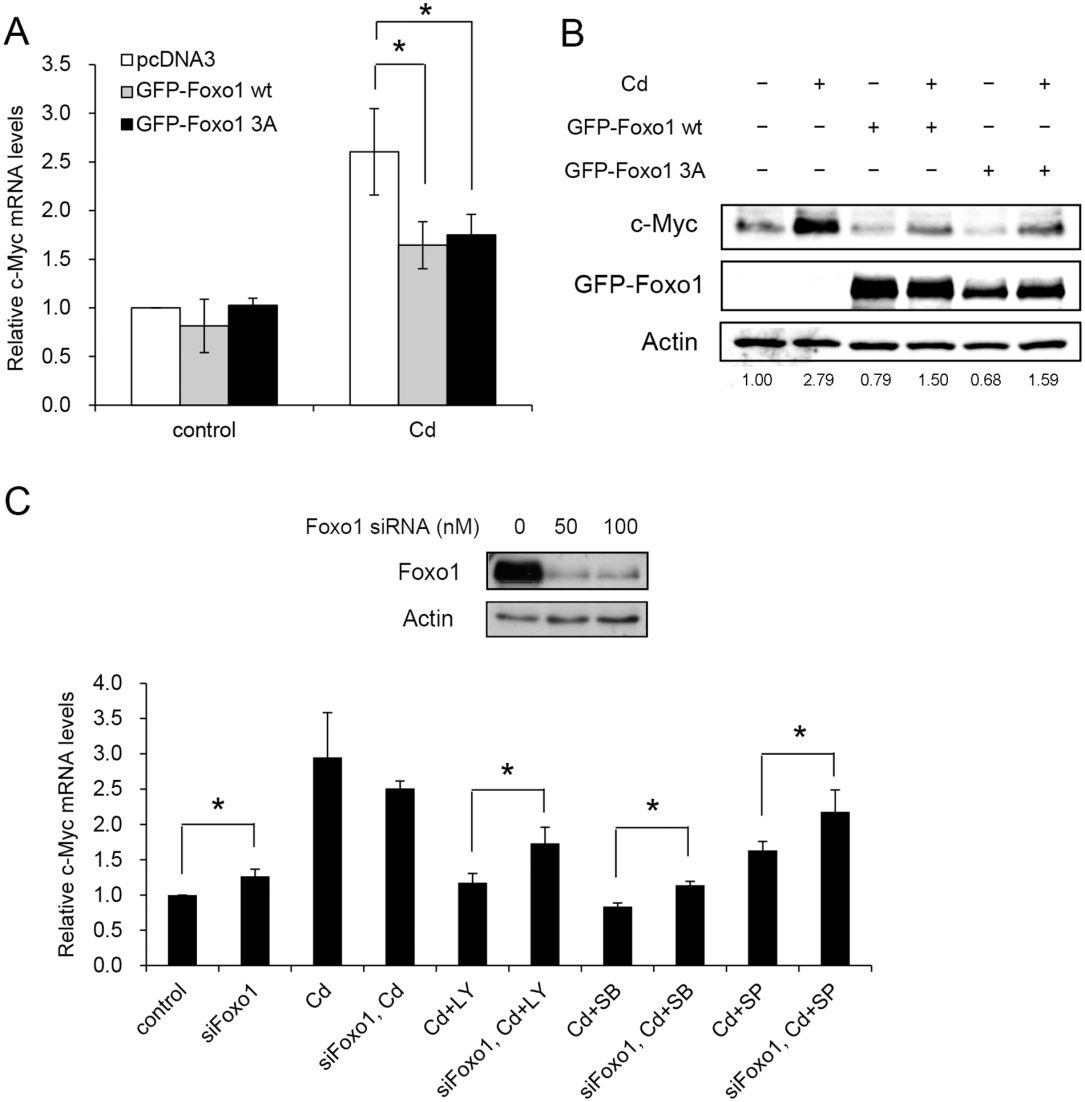
Cd-induced c-Myc expression is regulated by Foxo1. HepG2 cells were transfected with plasmid carrying wild type Foxo1 (GFP-Foxo1 WT) or constitutively active mutant (GFP-Foxo1 3A) gene. The cells were treated with 5 μM Cd for 4 h then harvested for c-Myc mRNA analysis by real-time PCR (A) and protein analyzed by Western blotting (B). Numbers underneath the Western blotting represent the amount of c-Myc relative to that of the loading control (actin). (C) Cells were transfected with 50 nM control or Foxo1 siRNA. Cells were cultured in the presence of 5 μM Cd for an additional 4 h before harvesting and cellular c-Myc mRNA quantified by real-time PCR. Upper panel of the figure shows the knock-down efficiency of the Foxo1 siRNA. For RNA analysis, each value represents a mean ± standard deviation of three samples. Asterisks (*) indicate significant differences (*p* < 0.05) between the paired samples. Actin was used as a loading control for immunoblotting.

The role of Foxo1 in c-Myc expression was examined. Cells were transfected with wild type Foxo1 or a mutant with alanines substituted at the three phosphorylation sites (Foxo1 3A; a constitutively active Foxo1) [38]. The c-Myc mRNA in Cd-treated cells had significant reduction at the transcriptional level (Fig 9A). However, no difference was noted in the c-Myc mRNA levels between cells expressing the wild type or the mutant Foxo1 gene. Similar observations were obtained at the protein level. Addition of Cd increased the c-Myc level, and it dropped markedly with the expression of wild type or the Foxo1 3A mutant (Fig 9B).

The above results show that increase of Foxo1 attenuates c-Myc expression. We then investigated the effect of reducing Foxo1 level on the c-Myc gene expression. Foxo1 expression was knocked-down by siRNA, followed by the addition of p38, JNK or PI3K inhibitor. Fig 9C shows that administration of various inhibitors reduces the Cd-induced c-Myc mRNA level. With the knock-down of Foxo1 expression, c-Myc mRNA elevated in the inhibitor-treated cells. Since Foxo1 does not act on the c-Myc promoter, the result indicates that expression of Foxo1 suppresses the quantity of c-Myc mRNA.

## Discussion

**Fig 10:**
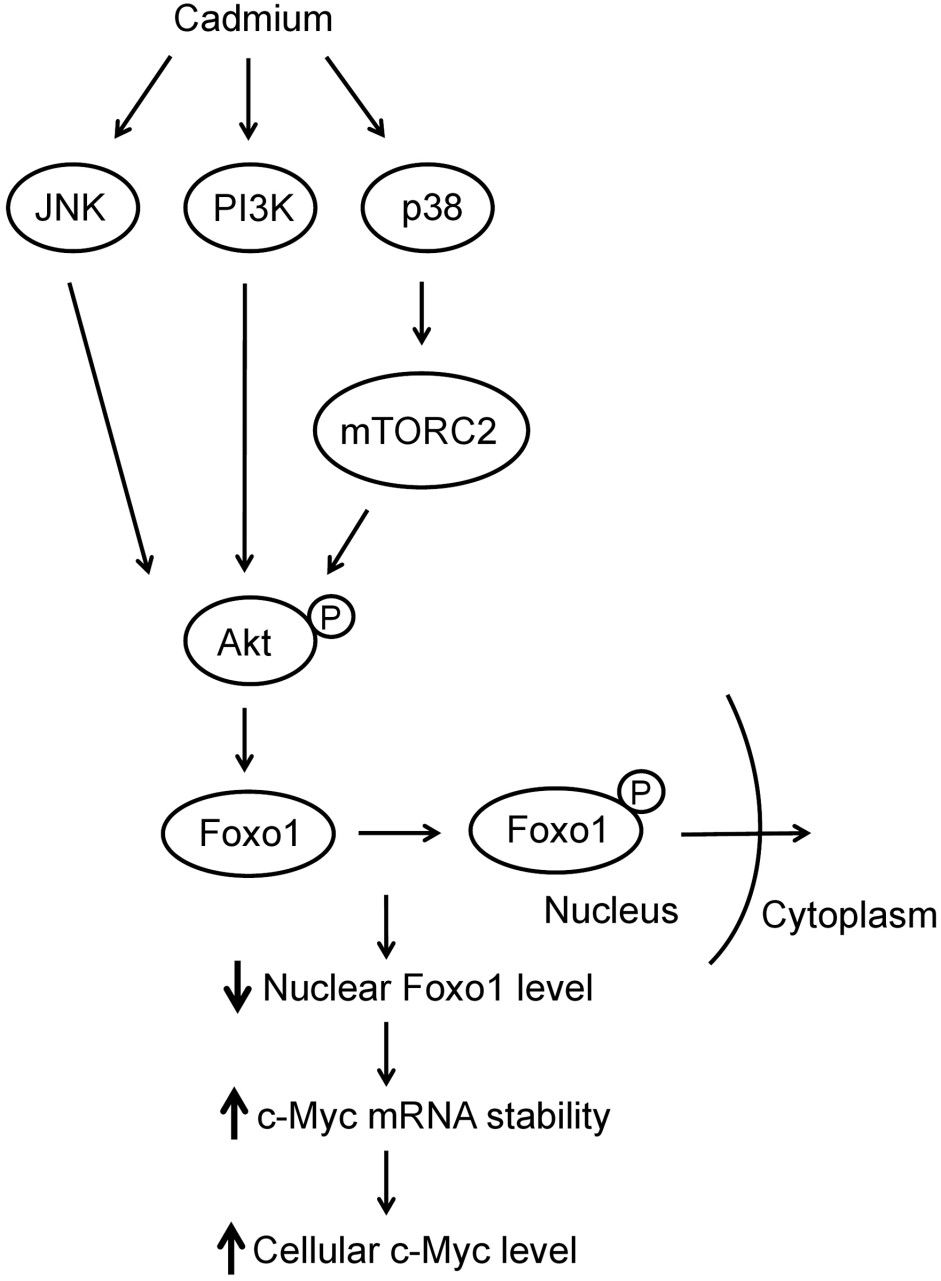
Schematic illustration of the mechanism associated with the Cd-induced elevation of c-Myc in HepG2 cells.

Cd is an environmental contaminant and also a carcinogen for human [1]. Cellular exposure to Cd may result in cell damage, cell death or carcinogenesis [39]. Reportedly, Cd induces the expression of several oncogenes, such as c-Jun, c-Fos and c-Myc, which may stimulate cell proliferation and leads to oncogenesis [40]. We investigate here the mechanism of Cd-induced c-Myc expression in hepatic cells. The elevation of c-Myc with Cd administration derives from enhancing the c-Myc mRNA stability (Fig 2C). Cd activates multiple signaling factors as examined in this study. All of the signaling factors examined here coordinately elevate the Akt activity. The functional Akt then phosphorylates Foxo1 and translocates the modified protein from the nucleus into the cytoplasm. The reduction of nuclear located Foxo1 leads to a decrease in its target gene that may affect the c-Myc mRNA stability. The postulated signaling pathway evoked by Cd treatment that causes the increase accumulation of cellular c-Myc is illustrated in Fig 10.

The signaling factor p38 can be activated by a variety of stresses. In turn, p38 may transduce the signal to different downstream pathways to cause various cellular responses [41–44]. Reportedly, Akt is a downstream target of p38 [45]. We found here that p38 activates Akt through mTORC2 in Cd-treated cells (Fig 4F). The signal linkage of p38/mTORC2/Akt has been noted in other studies. Under oxidative stress, Rit activates the p38/mRORC2/Akt pathway to attenuate H_2_O_2_-induced cell damage [46]. Possibly, p38 exerts its effect via Rictor, which is a component of the mTORC2 complex. Dominant negative p38 or p38 inhibitor (SB202190) has been shown to reduce the assembly capacity of mTORC2 in rat hepatic cells [47]. The assembly of mTORC2 complex requires the phosphorylation of Rictor [48]. Therefore, p38 is expected to phosphorylate Rictor in the Cd-induced signaling pathway.

Activation of JNK is frequently associated with stimulation of cell death or inhibition of tumor growth. However, contradictory results such as JNK activation enhances cell proliferation or tumor formation have also been reported [49]. Akt is a downstream effector of JNK. We show here that JNK activates Akt in Cd-treated cells and leads to the elevation of the c-Myc level. Studies indicate that Akt can be phosphorylated at Thr450 by JNK to attain basal activity. Further modifications at Thr308 and Ser473 allow the Akt to gain integral activity [37]. The activated Akt then transduces the signal to a variety of downstream effectors. However, differential modification of Thr308 or Ser473 may result in the activation of different downstream targets. Knockout of Rictor expression abolishes the mTORC2 activity and Ser473 of Akt was not modified under insulin stimulation. Examining the downstream targets of Akt under this situation reveals that Foxo3 activity was inhibited but the phosphorylations at TSC2 and GSK3 were not affected [50]. These results indicate that phosphorylation of Akt at specific sites may regulate different cell activities. Untreated HepG2 cells have Thr450 modification and Cd administration did not alter the level of this modification. However, Cd treatment increases Thr308 and Ser473 phosphorylation. Addition of JNK inhibitor reduced not only the basal (Thr450) but also the induced (Thr308 and Ser473) modifications (Fig 5). This result indicates that JNK maintains basal Akt activity which is required for subsequent Thr308 and Ser473 phosphorylations to achieve full activity after Cd stimulation. JNK inhibitor reduces Thr450 modification and thus Thr308 and Ser473 cannot be effectively modified with Cd treatment. Noticeably, PI3K and p38 inhibitor can also concurrently reduce the phosphorylation of these three sites (Fig 5). If phosphorylation of Thr450 is a prerequisite for the modification of Thr308 and Ser473, then both PI3K and p38 signals apparently contribute also to the modification of Akt at Thr450.

Foxo proteins are transcriptional factors. Phosphorylation of Foxo by Akt allows the modified protein to interact with 14-3-3. The complex then translocates from the nucleus to the cytoplasm and reduces the transcriptional activity of Foxo [24]. We found that PI3K inhibitor, Rictor siRNA and p38 inhibitor effectively reduced the Cd-induced phosphorylation and the translocation of Foxo1 (Figs 7 and 8). Therefore, PI3K, Akt, mTORC2 and p38 are upstream regulators of Foxo1. However, treating cells with JNK inhibitor attenuates Cd-induced phosphorylation of Foxo1 but does not alter significantly its translocation (Figs 7A and 8D). Variation in cellular localization of Foxo protein by JNK has been documented. Reportedly, JNK can phosphorylate Foxo4 at Thr447 and/or Thr451 and leads to its translocation from the cytoplasm to the nucleus [51]. The JNK-induced modification does not interfere with the Foxo4 phosphorylation by Akt and its interaction with 14-3-3. Foxo1 might behave similarly in the presence of JNK and needs further investigation. It has been suggested that the balance between JNK and the Akt activities can determine the cellular localization of Foxo [51, 52]. With a stronger JNK activity, Foxo moves into nucleus and exerts its transcription activity. On the contrary, with a stronger Akt activity, Foxo translocates into cytoplasm and losses its activity. We speculate that JNK activity may enhance the modification of Akt and then phosphorylate the Foxo1 in Cd-treated cells. However, the JNK activity alone is not sufficient to drive the Foxo1 back to the nucleus. Therefore, Foxo1 level did not increase in nucleus even though the JNK inhibitor reduced Foxo1 phosphorylation (Figs 7A and 8D).

Activation of Foxo can antagonize the function of c-Myc. Foxo increases the levels of repressors that target the c-Myc gene and thus reduces its expression [53]. Additionally, Foxo can modulate c-Myc mRNA stability or translation by altering the level of miR-145 or miR-34c [19]. As shown in this study, PI3K, p38 or JNK inhibitor reduced the amount of cellular c-Myc mRNA (Figs 3B and 9C). Addition of Foxo1 siRNA with inhibitors can partially recover the c-Myc mRNA level (Fig 9C). Therefore, Foxo1 plays an inhibitory role in regulating the c-Myc expression, and it is understream of PI3K, p38 or JNK on the signaling pathway.

The promoter of the c-Myc gene does not have Foxo1 binding site. Therefore, Foxo1 does not directly regulate the c-Myc gene expression. Since Cd enhances the stability of c-Myc mRNA (Fig 2C), factors relating to this effect were explored. Several factors such as HuR, CRD-BP and TTP (tristetraprolin) that bind the AU-rich element at the 3’-UTR or coding sequence of c-Myc mRNA were studied. However, none of their expression is associated with the c-Myc mRNA stability in Cd-treated cells (data not shown). Additionally, the role of microRNA was also analyzed. We found that miR-145 expression was not altered by Cd treatment, but quantification of miR-34c in HepG2 cells was not successful. We therefore constructed a reporter plasmid harboring the miR-34c promoter region to study the regulatory mechanism. The miR-34c promoter has two Foxo binding sites. Administration of Cd reduced the reporter activity (S3 Fig). However, further addition of inhibitors did not recover the reporter activity except JNK inhibitor. We also constructed a reporter plasmid by fusing luciferase gene with the 3’-UTR of c-Myc mRNA. The reporter activity did not change after Cd treatment (data not shown). Base on this finding, we cannot interpret whether miR-34c is participated in the regulation of c-Myc mRNA after Cd exposure.

Long-term culture in medium containing low concentration of Cd allows cells to gain more resistance to Cd toxicity. MT, γ-GCSh and GSH expressions are increased in these cells and contribute to the elevation of Cd tolerance [54]. However, this adaptive process might lead cells to carcinogenesis since Cd is known to increase the c-Myc level [5]. Noticeably, c-Myc regulates γ-GCSh expression to alter the cellular GSH content and Cd tolerance [27]. Since c-Myc is a proto-oncogene, the expression of c-Myc might increase the viability of Cd-damaged cells under Cd challenge. The proto-oncogene might also accelerate the un-repaired or mis-repaired cells to the oncogenic process. We report here that Cd stimulates c-Myc expression by increasing the stability of c-Myc mRNA. The increase in c-Myc level is achieved through the activation of multiple signaling factors to coordinately activate the Akt/Foxo1 pathway. Cd induces a variety of signaling factors which are not necessary associated with the c-Myc expression (such as ERK). The activated PI3K, p38 and JNK can transduce the signal to other pathways in addition to Akt. All of these factors may play different roles to determine the cell fate after Cd exposure.

## Supporting Information

S1 Fig Effect of Rictor siRNA knock-down on the c-Myc mRNA stability in Cd-treated cells. Cells were transfected with 50nM control or Rictor siRNA. After adding 0 or 5 μM Cd for 2 h, actinomycin D (5 μg/ml) was added. Cells were cultured for additional 2 h then harvested for analysis. Asterisks (*) indicate significant differences (*p* < 0.05) between the paired samples. Each value represents a mean ± standard deviation of three samples.
(TIF)

S2 Fig Activation of Akt in cytoplasmic and nuclear fractions of cells after Cd treatment. Cells were treated with 5 μM Cd for various time intervals. Extracts from cytosolic and nuclear fractions were prepared and phospho-Akt levels were determined by Western blotting.
(TIF)

S3 Fig Effect of Cd treatment on miR-34c promoter activity. HepG2 cells were transfected with a reporter plasmid carrying the miR-34c promoter region (-1630 to +27). Transfected cells were pretreated with 50 μM LY294002 (LY), SB202190 (SB) or SP600125 (SP) for 1 h, following by the addition of 5 μM Cd and culturing for 6 h. The luciferase activity of the cells was analyzed. Asterisks (*) indicate significant differences (*p* < 0.05) as compared to that of the control. Each value represents a mean ± standard deviation of three samples.
(TIF)

S1 Table Primer sequences for constructing a reporter plasmid and qPCR assays. (PDF)
