# Supplementary figures and images for: Cadmium Activates Multiple Signaling Pathways That Coordinately Stimulate Akt Activity to Enhance c-Myc mRNA Stability
Source: PLoS One. 2016 Jan 11;11(1):e0147011. doi: 10.1371/journal.pone.0147011 (PMC4709241; doi:10.1371/journal.pone.0147011)

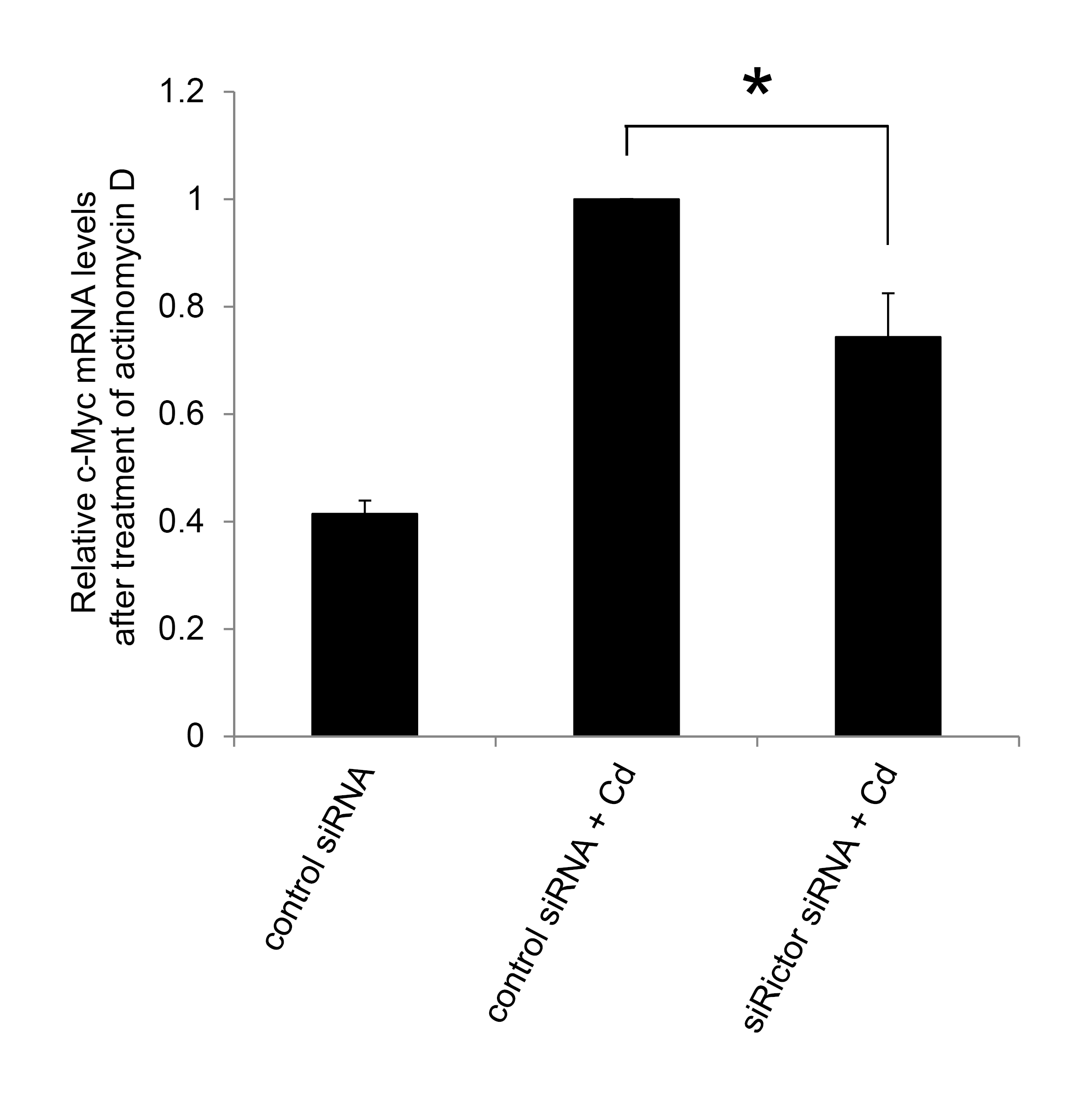

Supplement: S1 Fig — Cells were transfected with 50nM control or Rictor siRNA. After adding 0 or 5 μM Cd for 2 h, actinomycin D (5 μg/ml) was added. Cells were cultured for additional 2 h then harvested for analysis. Asterisks (*) indicate significant differences (p < 0.05) between the paired samples. Each value represents a mean ± standard deviation of three samples. (TIF) [file pone.0147011.s001.tif]

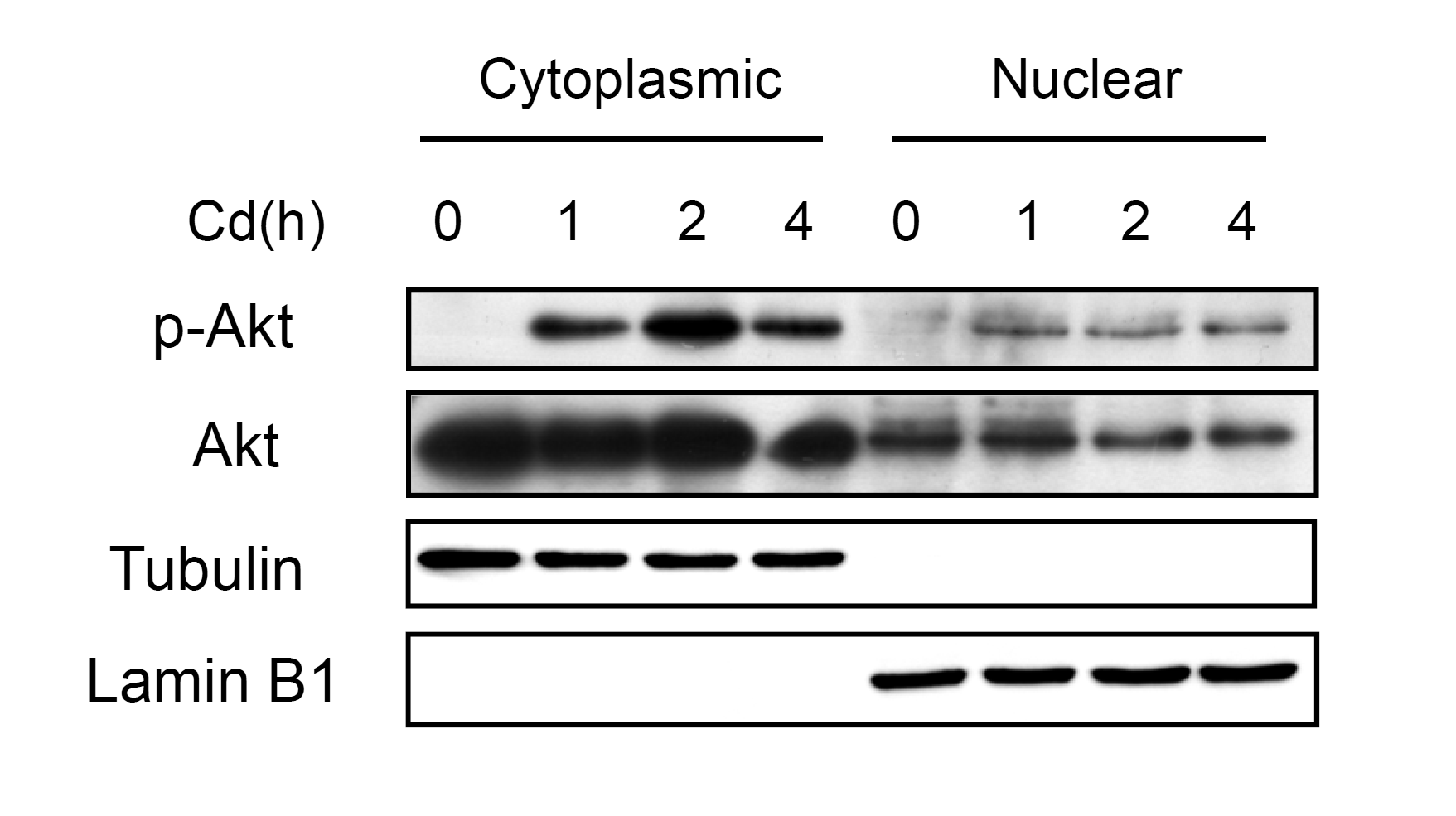

Supplement: S2 Fig — Cells were treated with 5 μM Cd for various time intervals. Extracts from cytosolic and nuclear fractions were prepared and phospho-Akt levels were determined by Western blotting. (TIF) [file pone.0147011.s002.tif]

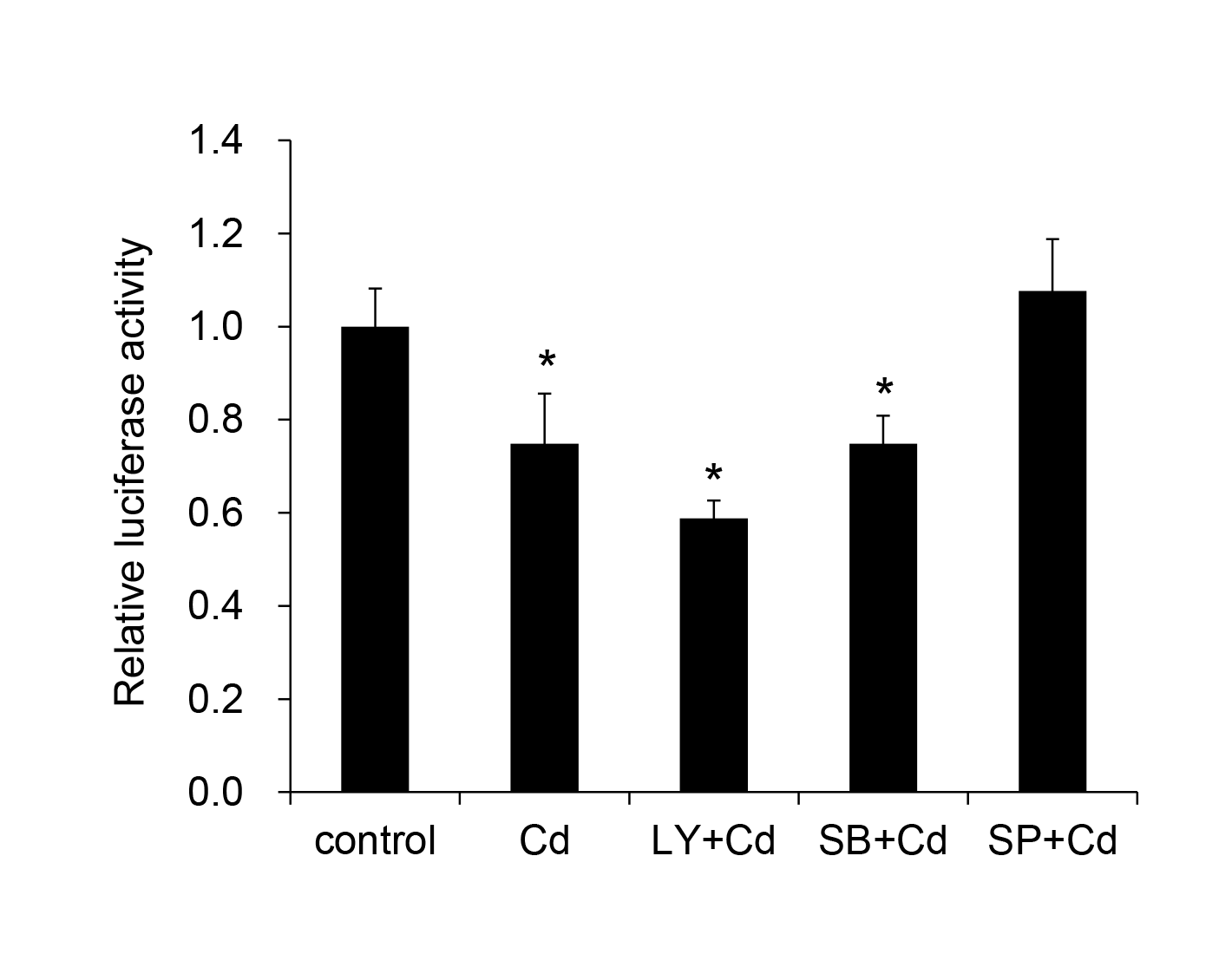

Supplement: S3 Fig — HepG2 cells were transfected with a reporter plasmid carrying the miR-34c promoter region (-1630 to +27). Transfected cells were pretreated with 50 μM LY294002 (LY), SB202190 (SB) or SP600125 (SP) for 1 h, following by the addition of 5 μM Cd and culturing for 6 h. The luciferase activity of the cells was analyzed. Asterisks (*) indicate significant differences (p < 0.05) as compared to that of the control. Each value represents a mean ± standard deviation of three samples. (TIF) [file pone.0147011.s003.tif]
