# Supplementary material for: Cadmium Activates Multiple Signaling Pathways That Coordinately Stimulate Akt Activity to Enhance c-Myc mRNA Stability
Source: PLoS One. 2016 Jan 11;11(1):e0147011. doi: 10.1371/journal.pone.0147011 (PMC4709241; doi:10.1371/journal.pone.0147011)
Supplement: S1 Table — (PDF) [file pone.0147011.s004.pdf]

S1 Table. Primer sequences for constructing a reporter plasmid and qPCR assays.

**Name**	**Primer sequences (5'-3')**
**Primers to construct a reporter plasmid**	
c-Myc promoter	Forward : ATGTAGATCTCTCCCGTCTAGCACCTTTG
	Reverse : CTCTGCCTCTCCATGGAATTACTACAGCG
**qPCR primers**	
c-Myc	Forward : GCCACGTCTCCACACATCAG
	Reverse : TGGTGCATTTTCGGTTGTTG
GAPDH	Forward : GAAGGTGAAGGTCGGAGTC
	Reverse : GAAGATGGTGATGGGATTTC
